# ciRS-7 is a prognostic biomarker and potential gene therapy target for renal cell carcinoma

**DOI:** 10.1186/s12943-021-01443-2

**Published:** 2021-11-05

**Authors:** Weipu Mao, Keyi Wang, Bin Xu, Hui Zhang, Si Sun, Qiang Hu, Lei Zhang, Chunhui Liu, Shuqiu Chen, Jianping Wu, Ming Chen, Wei Li, Bo Peng

**Affiliations:** 1 Department of Urology, Affiliated Zhongda Hospital of Southeast University, No. 87 Dingjiaqiao, Hunan Road, Gulou District, Nanjing, 210009 China; 2 Surgical Research Center, Institute of Urology, Southeast University Medical School, Nanjing, 210009 China; 3 Department of Urology, Nanjing Lishui District People’s Hospital, Zhongda Hospital Lishui Branch, Southeast University, Nanjing, 211200 China; 4 Department of Urology, Shanghai Tenth People’s Hospital, School of Medicine, Tongji University, No. 301, Yanchang Road, Jing’an District, Shanghai, 200072 China; 5 Department of Anesthesiology, Shanghai Tenth People’s Hospital, School of Medicine, Tongji University, Shanghai, 200072 China

**Keywords:** Renal cell carcinoma, ciRS-7, PBAE/si-ciRS-7 nanocomplexes, Metastasis, Gene therapeutic

## Abstract

Circular RNAs are a new class of non-coding RNAs that have been shown to play critical roles in the development and progression of renal cell carcinoma (RCC). However, little is known about the functional mechanisms and therapeutic role of ciRS-7 in RCC. A series of in vitro and in vivo experiments were performed to investigate the functional mechanism and therapeutic role of ciRS-7, such as real-time quantitative PCR, CCK-8, wound healing, transwell, colony formation, Edu, tumor xenograft and lung metastasis in NSG mice. RNA pull-down, dual luciferase reporter, fluorescence in situ hybridization (FISH) and rescue assays were used to determine the relationship between ciRS-7, miR-139-3p and TAGLN. In addition, we constructed PBAE/si-ciRS-7 nanocomplexes with PBAE material to evaluate the therapeutic effect of the nanocomplexes on tumor in vivo. ciRS-7 was highly expressed in RCC tumor tissues and cell lines, and high ciRS-7 expression correlated with tumor size, high Fuhrman grade and poor survival. Depletion of ciRS-7 significantly inhibited RCC cell proliferation, invasion, tumor growth and metastasis in vivo, while overexpression of ciRS-7 had the opposite effect. Mechanistically, ciRS-7 acts as a "ceRNA" for miR-139-3p to prevent TAGLN degradation and promoting RCC progression and metastasis via the PI3K/AKT signaling pathway. In addition, miR-139-3p mimics or inhibitor could reverse the altered malignant tumor behavior caused by ciRS-7 overexpression or silencing. Furthermore, the PBAE/siciRS-7 nanocomplexes could significantly inhibit RCC tumor progression and metastasis in vivo. ciRS-7 acts as a tumor promoter by regulating the miR-139-3p/TAGLN axis and activating the PI3K/AKT signaling pathway to promote RCC progression and metastasis. Drug development of PBAE/si-ciRS-7 nanocomplexes targeting ciRS-7 may represent a promising gene therapeutic strategy for RCC.

**Supplementary Information:**

The online version contains supplementary material available at 10.1186/s12943-021-01443-2.

## Background

Renal cell carcinoma (RCC) is a common malignant tumor of the urinary system, only second to prostate and bladder cancers in incidence, which has been on a continuous rise in the last decade [1–3]. Since RCC is insensitive to radiotherapy and chemotherapy, gene-targeted therapy has provided a promising new direction for its treatment; several targeted drugs are now being used in clinical practice [4]. Circular RNAs (circRNAs) are a newly discovered type of non-coding RNAs (ncRNAs). Accumulating evidence suggests that circRNAs may play a crucial role in the pathogenesis of various malignancies, including RCC, bladder cancer, breast cancer, and hepatocellular carcinoma [5–7]. However, the functional mechanisms underlying the therapeutic roles of ciRS-7 in RCC remain unknown.

## Results

### ciRS-7 is overexpressed in RCC cells and tissues and it promotes in vitro cell proliferation, migration and invasion

**Fig. 1:**
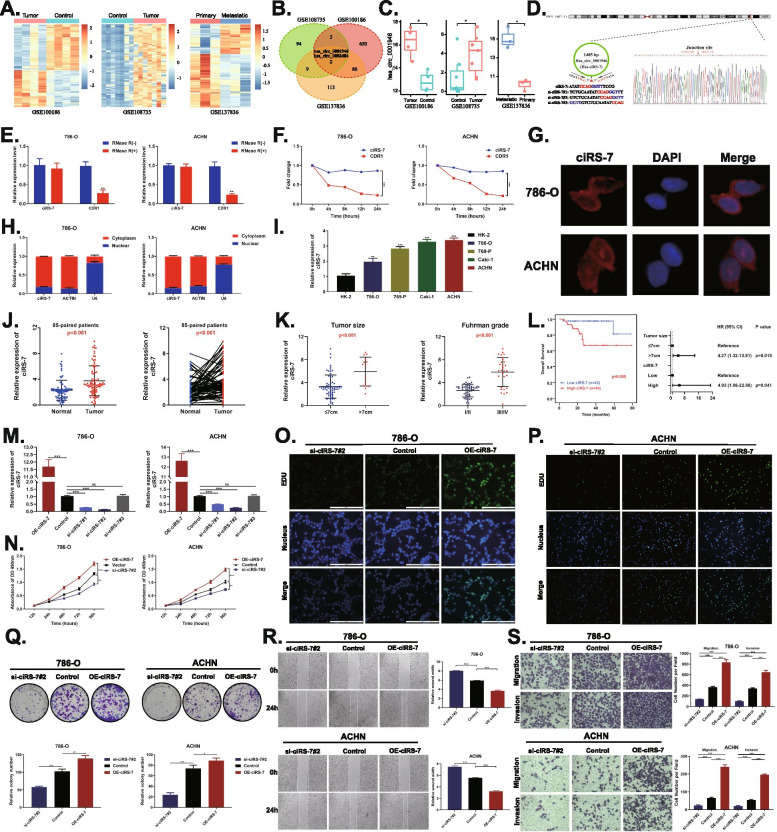
ciRS-7 is overexpressed in RCC tissues and cells and promotes RCC cell proliferation, migration and invasion in vitro*.* **A**, Hierarchical clustering analysis of differentially expressed circRNAs in GSE100186, GSE108735 and GSE137836. **B**. Venn diagram of circRNAs commonly highly expressed in GSE100186, GSE108735 and GSE137836. **C**. Expression of ciRS-7 in GSE100186, GSE108735 and GSE137836. **D**. Sanger sequencing to verify the splice junctions of ciRS-7. **E** and **F**. qRT-PCR analysis of ciRS-7 and linear CDR1 in 786-O and ACHN cells treated with RNase R or actinomycin D. **G**. FISH assay to detect the cellular localization of ciRS-7. **H**. qRT-PCR analysis of ciRS-7 was conducted in nuclear and cytoplasmic fractions of 786-O and ACHN cells. **I** and **J**. ciRS-7 was highly expressed in RCC cell lines and tumour tissues. **K**. Relative expression levels of ciRS-7 in different tumor sizes and Fuhrman grade. **L**. Kaplan-Meier’s survival curves showed the correlations between ciRS-7 expression and OS, and multivariate Cox regression of hazard ratios for RCC OS. **M.** Relative expression of ciRS-7 was confirmed by qPCR in 786-O and ACHN cell lines transfected with OE-ciRS-7, control, si-ciRS-7#1, si-ciRS-7#2 or si-ciRS-7#3. **N**. Growth curves of 786-O and ACHN cell lines were measured after transfection with indicated vectors by CCK-8. **O** and **P**. Edu assay to detect cell proliferation capacity after transfection with the indicated vectors. **Q**, Colony formation assay to detect cell migration ability after transfection with the indicated vectors. **R**. Wound healing assay to detect cell migration ability after transfection with the indicated vectors. **S.** Transwell assay to detect cell migration and invasion ability after transfection with the indicated vectors. (*p < 0.05, **p < 0.01, ***p < 0.001)

To identify circRNAs involved in RCC progression and metastasis, we obtained three GSE datasets (GSE100186, GSE108735, and GSE137836) from the GEO database. The Venn diagram showed that only two circRNAs (hsa_circ_0001946 and hsa_circ_0002484) were detected in three datasets and these were highly expressed in the tumor and metastatic tissues (Fig. 1A-C and Fig. S1A). For this study, we selected ciRS-7 (hsa_circ_0001946). ciRS-7 is formed due to the reverse shearing of CDR1. The ciRS-7 PCR product and its sequence were confirmed by agarose gel electrophoresis and Sanger sequencing, respectively (Fig. 1D and Fig. S1B). RNase R and actinomycin D assay showed that ciRS-7 was more stable than CDR1 (Fig. 1E, F). qRT-PCR and FISH showed that ciRS-7 was predominantly localized in the cytoplasm (Fig. 1G, H). We examined the expression of ciRS-7 in different cell lines and 85 pairs of tissues. The results showed that ciRS-7 was highly expressed in RCC tumor cell lines and tumor tissues (Fig. 1J, K). In addition, higher ciRS-7 expression was associated with greater tumor size, high Fuhrman grade, and poorer survival (Fig. 1L and Table S4, S5).

Three siRNAs (si-ciRS-7#1, si-ciRS-7#2 and si-ciRS-7#3) specifically targeting the junction sites of ciRS-7 and one overexpression lentivirus (OE-ciRS-7) were designed. qRT-PCR assay showed that si-ciRS-7#1 and si-ciRS-7#2 could significantly reduce the expression level of ciRS-7, while si-ciRS-7#3 had no significant knockdown effect on ciRS-7 (Fig. 1M). In addition, si-ciRS-7#2 had no effect on the expression level of the parental gene CDR1 (Fig. S1C). FISH showed that silencing of ciRS-7 indeed suppressed ciRS-7 expression in the cytoplasm in 786-O and ACHN cells; overexpression of ciRS-7 concomitantly increased its expression in the cytoplasm (Fig. S1C). CCK-8, colony formation, and EdU assays showed that inhibition of ciRS-7 could significantly attenuate the proliferation of 786-O and ACHN cells; converse was true for ciRS-7 overexpression (Fig. 1N-Q). In addition, wound healing and trans-well assays showed that the silencing of ciRS-7 could decrease the migration and invasion of 786-O and ACHN cells, while overexpression of ciRS-7 enhanced the migration and invasion of these cells (Fig. 1R, S).

### ciRS-7 acts as a sponge of miR-139-3p in RCC cells and miR-139-3p inhibits RCC cell proliferation, migration, and invasion in vitro

Since circRNAs which are localized in the cytoplasm generally exhibit miRNAs sponging, we further explored this possibility by predicting the potential miRNAs that could interact with ciRS-7 using circBank, miRanda, circAtlas, and RNAhybrid software. Two potential target miRNAs (miR-7-5p and miR-139-3p) were found to interact with ciRS-7 (Fig. S2A). Biotin-labeled ciRS-7 probing showed that both miR-7-5p and miR-139-3p were significantly pulled down by the ciRS-7 probe as compared to the NC probe (Fig. S2B). Using the TCGA KIRC database, we found that miR-7-5p was highly expressed in RCC tumor tissues, while miR-139-3p was significantly down-regulated (Fig. S2C). The dual-luciferase assays showed that ciRS-7 could interact with miR-139-3p at the third predicted site (Fig. S2D, E). In addition, RNA pull-down experiments showed that miR-139-3p could indeed interact with ciRS-7 (Fig. S2F, G). The double FISH assays also showed that ciRS-7 and miR-139-3p were co-localized in the cytoplasm of 786-O and ACHN cells (Fig. S2H). Furthermore, the expression of ciRS-7 was negatively correlated with miR-139-3p in the clinical samples (Fig. S2I).

Using the TCGA KIRC database, we found that miR-139-3p was downregulated in tumor tissues (Fig. S3A, B), miR-139-3p expression was significantly correlated with gender, TNM stage, tumor grade, and clinical stage (Fig. S3C-H) in this cohort, and patients with lower miR-139-3p expression had worse survival (Fig. S3I-K and Table S6). In addition, we found lower miR-139-3p expression in RCC tumor tissues among the 85 pairs of clinical samples that were tested (Fig. S4A). qRT-PCR showed that both miR-139-3p-Mimic and miR-139-3p plasmids increased the expression level of miR-139-3p (Fig. S4B). Functionally, CCK-8, wound healing, colony formation, EdU, trans-well migration, and invasion assays showed that silencing miR-139-3p could significantly promote proliferation, migration, and invasion in 786-O and ACHN cells, while miR-139-3p overexpression diminished these effects (Fig. S4C-G).

### TAGLN is a target of ciRS-7, and ciRS-7 regulates the miR-139-3p/TAGLN axis, activates the PI3K/AKT signaling pathway, and promotes RCC cell proliferation, migration, and invasion

The sh-ciRS-7 and sh-NC 786-O stable transduction cell lines were sequenced and we found that only TAGLN was downregulated at both the transcriptional and protein levels (Fig. S5A-C and Fig. S6A and Excel S3). The dual-luciferase assay also showed that miR-139-3p could bind to TAGLN (Fig. S5D). In addition, Kyoto Encyclopedia of Genes and Genomes (KEGG) enrichment analysis showed that PI3K/AKT signaling pathway was significantly enriched (Fig. S5E-G, Fig. S6B, C and Excel S5, S6).

Western blotting showed that the protein levels of TAGLN, p-AKT, and p-PI3K significantly reduced after transfection with sh-ciRS-7 or miR-139-3p-Mimic; the levels increased after transfected with OE-ciRS-7 or miR-139-3p-Inhibitor (Fig. S7A, B). At the protein level, we found that miR-139-3p-Mimic could partially counteract the increased expression of TAGLN, p-AKT, and p-PI3K due to OE-ciRS-7 (Fig. S7C). miR-139-3p-Inhibitor could partially rescue the reduced expression of TAGLN, p-AKT, and p-PI3K suppressed by sh-ciRS-7 (Fig. S7D). In addition, the results of CCK-8, wound healing, colony formation, EdU, trans-well migration, and invasion assays were consistent with the above findings (Fig. S7E-I).

### ciRS-7 enhances in vivo RCC tumor growth and metastasis and PBAE/si-ciRS-7 nanocomplexes can inhibit these effects

**Fig. 2:**
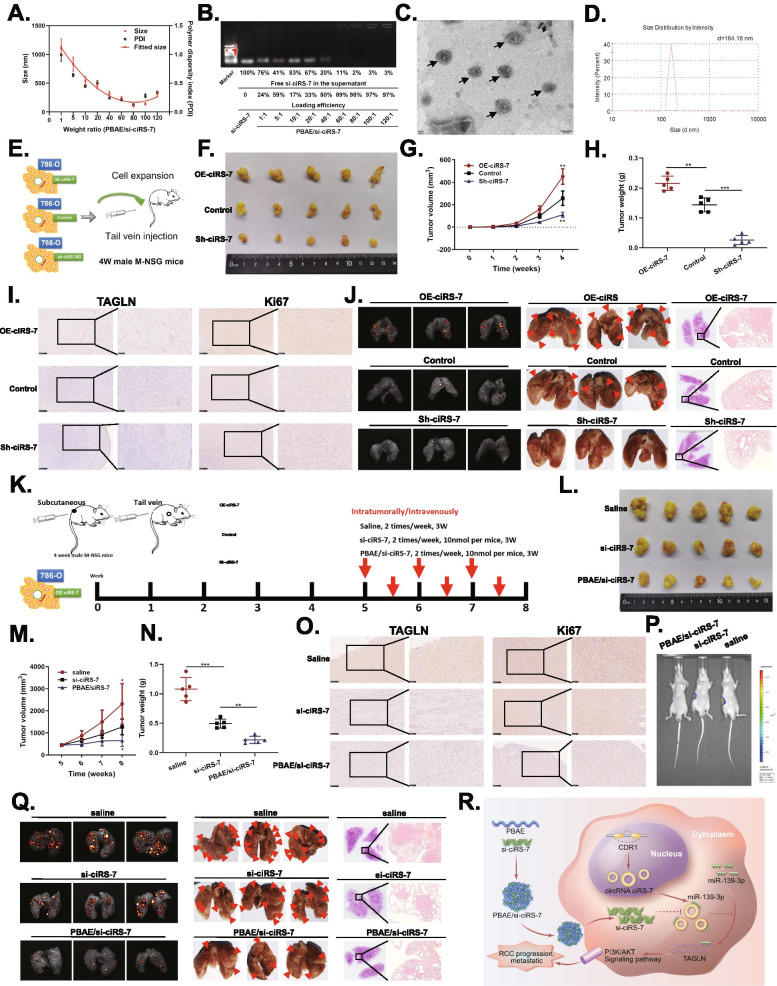
ciRS-7 enhances RCC tumor growth and metastasis in vivo and PBAE/si-ciRS-7 nanocomplexes inhibits RCC growth and metastasis in vivo*.* **A.** The sizes with different weight ratios of PBAE to siPlk1. The weight ratios of PBAE to si-ciRS-7 were ranged from 1 to 120. **B**. The loading efficiency of PBAE. **C**. TEM images of PBAE/si-ciRS-7 nanocomplexes (PBAE/si-ciRS-7 = 80/1). **D**. size distribution of PBAE/si-ciRS-7 nanocomplexes (PBAE/si-ciRS-7 = 80/1). **E.** Graphic illustration of the in vivo mice model study. **F-H**. 786-O cells stably transfected with OE-ciRS-7, control, or sh-ciRS-7 were injected subcutaneously into the mice. Tumor volume (**G**) and weight (**H**) increased in the OE-ciRS-7 group, while both tumor volume and weight decreased in the sh-ciRS-7 group. **I**. IHC assay demonstrated the level of TAGLN and Ki67 in pairs of tumours. **J**. After tail vein injection of treated cells, imaging, gross lung tissue lesions and HE staining were observed. **K.** The flow diagram showed the scheme of intratumorally/intravenously with saline, si-ciRS-7 or PBAE/si-ciRS-7 into mice. **L-N**. Weight volume and weight change after 3 weeks of treatment with saline, si-ciRS-7 or PBAE/si-ciRS-7 for xenograft tumors or lung metastasis models. **O**. IHC assay demonstrated the level of TAGLN and Ki67 in pairs of tumours. **P**. IVIS imaging of mice treated with saline, si-ciRS-7 or PBAE/si-ciRS-7 for subcapsular orthotopic implantation of the right kidney. **Q**. After tail vein injection of treated cells, imaging, gross lung tissue lesions and HE staining were observed. **R**. The hypothetical model depicts the roles of ciRS-7 in the promotion of RCC. (*p < 0.05, **p < 0.01, ***p < 0.001)

Based on the new insights into tumor pathogenesis, gene targeting therapy has gained attention as a potential approach for cancer treatment. Poly(β-amino ester) s (PBAEs) is an excellent candidate for gene delivery due to its low toxicity and high transfection efficiency [4, 8, 9]. The ^1^HNMR spectrum of PBAE is shown in Fig. S8A. We prepared nine nanocomplexes in different proportions according to the weight ratios of PBAE and si-ciRS-7. We found that relatively homogeneous nanoparticles were formed when the weight ratio of PBAE and si-ciRS-7 exceeded 40. Excellent performance and high loading efficiency (98%) were obtained for PBAE/si-ciRS-7 of 80 (Fig. 2A, B). Further both TEM and particle size potentiometer showed that the particle size of PBAE/si-ciRS-7 nanocomplexes was approximately 160 nm (Fig. 2C, D). In addition, the CCK-8 assay showed that PBAE/si-ciRS-7 nanocomplexes had a stronger inhibition of the proliferation of 786-O and ACHN cells as compared to PBAE and si-ciRS-7 alone (Fig. S8B, C).

To assess the effect of ciRS-7 on in vivo RCC growth and metastasis, we generated subcutaneous xenograft tumor and lung metastasis models (Fig. 2E). The xenograft tumor model showed that the tumor size and weight of mice in the OE-ciRS-7 group were significantly higher as compared to the control group, while the tumor size and weight of mice in the sh-ciRS-7 group were significantly lower (Fig. 2F-H). IHC staining showed that the expressions of Ki67 and TAGLN were significantly upregulated in the OE-ciRS-7 group and downregulated in the sh-ciRS-7 group (Fig. 2I).

In addition, the lung metastasis model showed that the OE-ciRS-7 group had more metastatic foci in the lungs of mice, followed by the control group, and the sh-ciRS-7 group had the least numbers. Micro-metastases were detected by H&E (Fig. 2J).

We further examined the effect of PBAE/si-ciRS-7 nanocomplexes on the in vivo RCC growth and metastasis (Fig. 2K). We found that the tumor size and weight of the mice in the PBAE/si-ciRS-7 nanocomplexes treated group was significantly reduced (Fig. 2L-N); IHC staining also showed a significant decrease in the expression of Ki67 and TAGLN (Fig. 2O). Moreover, IVIS imaging showed that PBAE/si-ciRS-7 nanocomplex injection minimized the size of renal in situ implantation tumors (Fig. 2P). In addition, in vivo lung metastasis models with PBAE/si-ciRS-7 nanocomplex treatment had better inhibition of lung metastasis (Fig. 2Q). The PBAE/si-ciRS-7 nanocomplexes could better inhibit the growth and metastasis of RCC tumors as compared to the animal-grade si-ciRS-7.

## Conclusion

As shown in Fig. 2R, this study showed that ciRS-7 could function as an oncogenic circRNA for the progression of RCC. ciRS-7 could promote RCC progression and metastasis through a novel regulatory pathway by binding miR-139-3p and blocking its inhibitory effect on TAGLN, which could promote RCC progression and metastasis through PI3K/AKT signaling pathway. In addition, we generated PBAE/si-ciRS-7 nanocomplexes, which, in the future, could provide novel insights for the development of RCC gene therapy-related drugs.

## Supplementary Information

**Additional file 1: Table S1.** Full sequence information of ciRS-7; **Table S2**. PCR primer, siRNA and probe sequence; **Table S3**. Antibodies list; **Table S4**. The relationship between the expression of ciRS-7 and various clinicopathological variables; **Table S5**. Univariate and multivariate Cox regression analysis and the relationship between ciRS-7 expression and overall survival; **Table S6**. Univariate and multivariate Cox regression analysis and the relationship between miR-139-3p expression and overall survival; **Figure S1**. ciRS-7 was overexpressed in RCC tissues. A. Volcano plots analysis of differentially expressed circRNAs in GSE100186, GSE108735 and GSE137836. B. Agarose gel electrophoresis of PCR products of ciRS-7 in RCC cell lines. C. Relative expression of linear CDR1 was confirmed by qPCR in 786-O and ACHN cell lines transfected with control and siciRS-7#2. D. Detection of ciRS-7 expression after transfection with the indicated vectors by FISH. Nuclei were stained blue (DAPI), ciRS-7 was stained red. **Figure S2**. ciRS-7 acts as a sponge of miR-139-3p in RCC cells. A. Two potential miRNAs absorbed by ciRS-7 were predicted through circBank, miRanda, circAtlas and RNAhybrid. B. Relative expression of two miRNAs enriched by ciRS-7 probe lysates was detected by qRT-PCR. C. Relative expression of two miRNAs in TCGA RCC database. D. Three possible binding sites of miR-139-3p to ciRS-7. E. Dual luciferase reporter assay demonstrated that miR-139-3p is a direct target of ciRS-7. F and G. RNA pull-down assay shown that miR-139-3p is a direct target of ciRS-7. H. Detection of colocalization of ciRS-7 and miR-139-3p in cytoplasm by RNA FISH assay. Nuclei were stained blue (DAPI), ciRS-7 was stained red, and miR-139-3p was stained green, I. Correlations between ciRS-7 and miR-139-3p expression were found with Pearson’s correlation analysis in RCC tissue samples (*n* = 85). (**p < 0.01).  **Figure S3**. miR-139-3p was downregulated in TCGA KIRC database. A. Expression of miR-139-3p in normal and tumors tissues of TCGA RCC dataset. B. Expression of miR-139-3p in normal and paired tumors tissues of TCGA RCC dataset. C-H. Relative expression levels of miR-139-3p in TCGA RCC subgroup: gender (C) tumor stage (D) lymphatic invasion (E) metastasis status (F) tumor grade (G) and tumor stage (H). I. Overall survival curve of RCC patients with low and high miR-139-3p expression. J and K. Univariate and multivariate cox regression analyses of miR-139-3p expression with overall survival in TCGA database. **Figure S4**. miR-139-3p inhibits RCC cell proliferation, migration and invasion in vitro. A. miR-139-3p had low expression in RCC tumour tissues compared with adjacent normal tissues. B. Relative expression of miR-139-3p was confirmed by qPCR in 786-O and ACHN cell lines transfected with miR-139-3p-NC, miR-139-3p-Mimic or pcDNA3.1/miR-139-3p. C. Growth curves of 786-O and ACHN cell lines were measured by CCK-8. D. Wound healing assay to detect cell migration ability. E. Transwell assay to detect cell migration and invasion ability. F. Colony formation assay to detect cell migration ability. G. Edu assay to detect cell proliferation capacity. (**p < 0.01, ***p < 0.001). **Figure S5**. TAGLN is a target gene of ciRS-7, and ciRS-7 activates the PI3K/AKT signaling pathway. A. Heatmap of RNA-Seq analysis of sh-NC and sh-ciRS-7 cells. Red in the heatmap denotes upregulation, blue denotes downregulation. B. Venn diagram showing the number of genes that changes at the transcriptional or protein levels. C. colloidal Coomassie detects changes in protein levels in 786-O and ACHN cells. D. Dual luciferase reporter assay demonstrated that TAGLN is a direct target of miR-139-3p. E-G. Enrichment chord plot (E), GO bubble plot (F) and KEGG enrichment histogram (G) of down-regulated proteins. (**p <0.01). **Figure S6**. Sequencing of sh-NC and sh-ciRS-7 cells. A. Heatmap of lab-free quantitative of sh-NC and sh-ciRS-7 cells. Red in the heatmap denotes upregulation, green denotes downregulation. B and C. GO bubble plot (B) and KEGG enrichment histogram (C) of up-regulated proteins. **Figure S7**. ciRS-7 regulating the miR-139-3p/TAGLN axis and activating the PI3K/AKT signaling pathway to promote RCC cell proliferation, migration and invasion. A. The expression of TAGLN, p-PI3K and p-AKT were detected by western blot after overexpression or knockdown of ciRS-7. B. The expression of TAGLN, p-PI3K and p-AKT were detected by western blot after overexpression or knockdown of miR-139-3p. C. Rescue assay of miR-139-3p after overexpression of ciRS-7 in 786-O cells. D. Rescue assay of miR-139-3p after knockdown of ciRS-7 in 786-O cells. E. Growth curves of 786-O and ACHN cell lines were measured by CCK-8. F. Colony formation assay to detect cell migration ability. G. Edu assay to detect cell proliferation capacity. H. Transwell assay to detect cell migration and invasion ability. I. Wound healing assay to detect cell migration ability. (*p < 0.05, **p < 0.01, ***p < 0.001). **Figure S8**. Characteristics of PBAE. A. The 1HNMR analysis of PBAE. B, C. Growth curves of 786-O and ACHN cell lines were measured by CCK-8.

**Additional file 2: Excel S1.** Differentially expressed circRNAs in GSE100186, GSE108735 and GSE137836.

**Additional file 3: Excel S2.** circBank, miRanda, circAtlas and RNAhybrid databases predict the possible miRNAs bound by ciRS-7.

**Additional file 4: Excel S3.** RNA sequencing results.

**Additional file 5: Excel S4.** Lab-free quantitative results.

**Additional file 6: Excel S5.** Down-regulation of the protein KEGG enrichment-related pathway.

**Additional file 7: Excel S6.** Up-regulation of the protein KEGG enrichment-related pathway.

## Data Availability

The datasets used and/or analyzed during the current study are available from the corresponding author on reasonable request.

## Abbreviations

RCC: Renal cell carcinoma
circRNA: Circular RNA
ncRNAs: Non-coding RNAs
PBAEs: Poly(β-amino ester)s
GEO: Gene Expression Omnibus
qRT-PCR: Quantitative real-time polymerase chain reaction
FISH: Fluorescence in situ hybridization
TEM: Transmission electron microscopy
H&E: Haematoxylin and eosin
IHC: Immunohistochemical
GAPDH: Glyceraldehyde-3-Phosphate Dehydrogenase
OS: Overall survival
