# Correction to: ciRS-7 is a prognostic biomarker and potential gene therapy target for renal cell carcinoma

**DOI:** 10.1186/s12943-021-01459-8

**Published:** 2021-12-01

**Authors:** Weipu Mao, Keyi Wang, Bin Xu, Hui Zhang, Si Sun, Qiang Hu, Lei Zhang, Chunhui Liu, Shuqiu Chen, Jianping Wu, Ming Chen, Wei Li, Bo Peng

**Affiliations:** 1 Department of Urology, Affiliated Zhongda Hospital of Southeast University, No. 87 Dingjiaqiao, Hunan Road, Gulou District, Nanjing, 210009 China; 2 Surgical Research Center, Institute of Urology, Southeast University Medical School, Nanjing, 210009 China; 3 Department of Urology, Nanjing Lishui District People’s Hospital, Zhongda Hospital Lishui Branch, Southeast University, Nanjing, 211200 China; 4 Department of Urology, Shanghai Tenth People’s Hospital, School of Medicine, Tongji University, No. 301, Yanchang Road, Jing’an District, Shanghai, 200072 China; 5 Department of Anesthesiology, Shanghai Tenth People’s Hospital, School of Medicine, Tongji University, Shanghai, 200072 China

**Correction to:** ***Mol Cancer*** **20, 142 (2021).**

**https://doi.org/10.1186/s12943-021-01443-2**

Following the publication of the original article [1], the authors found out that the Additional file 1 is outdated. Updated version is contained on this article, see Additional file section.

The original article has been corrected.

## Supplementary Information

**Additional file 1.**
