# Supplementary material for: ciRS-7 is a prognostic biomarker and potential gene therapy target for renal cell carcinoma
Source: Mol Cancer. 2021 Nov 5;20:142. doi: 10.1186/s12943-021-01443-2 (PMC8570002; doi:10.1186/s12943-021-01443-2)
Supplement: Supplementary file 1 — Additional file 1: Table S1. Full sequence information of ciRS-7; Table S2. PCR primer, siRNA and probe sequence; Table S3. Antibodies list; Table S4. The relationship between the expression of ciRS-7 and various clinicopathological variables; Table S5. Univariate and multivariate Cox regression analysis and the relationship between ciRS-7 expression and overall survival; Table S6. Univariate and multivariate Cox regression analysis and the relationship between miR-139-3p expression and overall survival; Figure S1. ciRS-7 was overexpressed in RCC tissues. A. Volcano plots analysis of differentially expressed circRNAs in GSE100186, GSE108735 and GSE137836. B. Agarose gel electrophoresis of PCR products of ciRS-7 in RCC cell lines. C. Relative expression of linear CDR1 was confirmed by qPCR in 786-O and ACHN cell lines transfected with control and siciRS-7#2. D. Detection of ciRS-7 expression after transfection with the indicated vectors by FISH. Nuclei were stained blue (DAPI), ciRS-7 was stained red. Figure S2. ciRS-7 acts as a sponge of miR-139-3p in RCC cells. A. Two potential miRNAs absorbed by ciRS-7 were predicted through circBank, miRanda, circAtlas and RNAhybrid. B. Relative expression of two miRNAs enriched by ciRS-7 probe lysates was detected by qRT-PCR. C. Relative expression of two miRNAs in TCGA RCC database. D. Three possible binding sites of miR-139-3p to ciRS-7. E. Dual luciferase reporter assay demonstrated that miR-139-3p is a direct target of ciRS-7. F and G. RNA pull-down assay shown that miR-139-3p is a direct target of ciRS-7. H. Detection of colocalization of ciRS-7 and miR-139-3p in cytoplasm by RNA FISH assay. Nuclei were stained blue (DAPI), ciRS-7 was stained red, and miR-139-3p was stained green, I. Correlations between ciRS-7 and miR-139-3p expression were found with Pearson’s correlation analysis in RCC tissue samples (n = 85). (**p < 0.01). Figure S3. miR-139-3p was downregulated in TCGA KIRC database. A. Expression of miR-139-3p in nor [file 12943_2021_1443_MOESM1_ESM.docx]

**ciRS-7 is a Prognostic Biomarker and Potential Gene Therapy Target for Renal Cell Carcinoma**

Weipu Mao, Keyi Wang, Bin Xu, Hui Zhang, Si Sun, Qiang Hu, Lei Zhang^1^ Chunhui Liu, Shuqiu Chen, Jianping Wu, Ming Chen, Wei Li, Bo Peng

Supplementary Information

Materials and Methods-----------------------------------------------------------------------------------3

Reference-------------------------------------------------------------------------------------------------11

**Table S1.** Full sequence information of ciRS-7------------------------------------------------------12

**Table S2.** PCR primer, siRNA and probe sequence-------------------------------------------------13

**Table S3.** Antibodies list--------------------------------------------------------------------------------14

**Table S4**. The relationship between the expression of ciRS-7 and various clinicopathological variables--------------------------------------------------------------------------------------15

**Table S5.** Univariate and multivariate Cox regression analysis and the relationship between ciRS-7 expression and overall survival---------------------------------------------------16

**Table S6.** Univariate and multivariate Cox regression analysis and the relationship between miR-139-3p expression and overall survival---------------------------------------------17

**Fig S1**. ciRS-7 was overexpressed in RCC tissues--------------------------------------------------18

**Fig S2**. ciRS-7 acts as a sponge of miR-139-3p in RCC cells--------------------------------------19

**Fig S3**. miR-139-3p was downregulated in TCGA KIRC database-------------------------------20

**Fig S4**. miR-139-3p inhibits RCC cell proliferation, migration and invasion *in vitro*----------21

**Fig S5**. TAGLN is a target gene of ciRS-7, and ciRS-7 activates the PI3K/AKT signaling pathway------------------------------------------------------------------------------------------22

**Fig S6.** Sequencing of sh-NC and sh-ciRS-7 cells---------------------------------------------------23

**Fig S7**. ciRS-7 regulating the miR-139-3p/TAGLN axis and activating the PI3K/AKT signaling pathway to promote RCC cell proliferation, migration and invasion---------24

**Fig S8**. Characteristics of PBAE-----------------------------------------------------------------------25

**Excel S1.** Differentially expressed circRNAs in GSE100186, GSE108735 and GSE137836.

**Excel S2.** circBank, miRanda, circAtlas and RNAhybrid databases predict the possible miRNAs bound by ciRS-7.

**Excel S3.** RNA sequencing results.

**Excel S4.** Lab-free quantitative results.

**Excel S5.** Down-regulation of the protein KEGG enrichment-related pathway.

**Excel S6.** Up-regulation of the protein KEGG enrichment-related pathway.

**Materials and Methods**

**Clinical specimens**

A total of 85 pairs of RCC tumor tissues and their corresponding adjacent normal renal tissues from patients who underwent nephrectomy at the Shanghai Tenth People's Hospital of Tongji University (Shanghai, China) and the Zhongda Hospital of Southeast University (Nanjing, China) between January 2014 and December 2019, were collected. Patient demographics and clinicopathological information are shown in **Table 1**. The pathology of all the RCC patients was confirmed by the pathologists of the hospitals; the pathological staging was determined according to the American Joint Committee on Cancer TNM staging system (7th edition). ciRS-7 expression levels were compared with the mean values. Expression of ciRS-7 was defined based on its average as high or low when the values were greater or lower than the average, respectively. The study design was evaluated and approved by the Ethics Committee of both the Shanghai Tenth People's Hospital and the Zhongda Hospital of Southeast University. All experiments were performed following the regulations of the Ethics Committee of the Shanghai Tenth People's Hospital (SHSY-IEC-BG/02.04/04.0-81602469). Written informed consent was obtained from all the patients or their relatives.

**Cell lines and cell culture**

Human RCC cell lines 786-O, 769-P, Caki-1, and ACHN and normal renal tubular epithelial cells, HK-2 were purchased from the Cell Bank of the Chinese Academy of Sciences (Shanghai, China). 786-O, 769-P, and Caki-1 cells were cultured in Dulbecco's modified Eagle's medium (DMEM, Gibco; Thermo Fisher Scientific, USA), ACHN cells in RPMI-1640 medium (Gibco; Thermo Fisher Scientific, USA), and HK-2 cells in keratinocyte medium (KM, ScienCell, USA) supplemented with 1% keratinocyte growth supplement (KGS, ScienCell, USA). All media were supplemented with 10% fetal bovine serum (FBS, Gibco; Thermo Fisher Scientific, USA) and 1% penicillin/streptomycin (Gibco; Thermo Fisher Scientific, USA) and the cells were cultured at 37°C in a humidified incubator with 5% CO2. RCC cell lines were stored at -80°C using CELLSAVING reagent (NCM, Suzhou, China).

**Cell transfection**

Three small interfering RNAs specifically targeting ciRS-7 (si-ciRS-7 #1, si-ciRS-7 #2 and si-ciRS-7 #3), siRNA for negative control (Control), miR-139-3p mimics (miR-139-3p-Mimics), miR-139-3p control (miR-139-3p-NC), and miR-139-3p inhibitor (miR-139-3p-Inhibitor), were purchased from RiboBio (Guangzhou, China). In addition, the plasmid for miR-139-3p was constructed by Integrated (Shanghai, China). In order to design effective siRNAs targeting to ciRS-7, we designed siRNAs against the ciRS-7 junction site. The sequence of ciRS-7 junction site is ATATCCAGGGTTTCCG. si-ciRS-7#1 sequence: TCTGCAATATCCAGGGTTT; si-ciRS-7#2 sequence: GTCTGCAATATCCAGGGTT; si-ciRS-7#3 sequence: GGTTGTCTGCAATATCCAG. si-ciRS-7#3 is the mutant sequence of si-ciRS-7#2. The difference between si-ciRS-7#3 and si-ciRS-7#2 is the base order in the sequences. We spliced the base sequence (GGTT) on the junction side of the si-ciRS-7#2 sequence to the 5' end (GTCTGCAATATCCAG) to form si-ciRS-7#3 (**Fig. 1D**). Transient transfection was performed at 30%-50% cell confluence using Lipofectamine 3000 (Thermo Fisher Scientific, USA). ciRS-7 knockdown lentivirus (sh-ciRS-7) containing the si-ciRS-7, and ciRS-7 overexpressing lentivirus (OE-ciRS-7) were synthesized by Genomeditech (Shanghai, China).

**Expression profile analysis of circRNAs**

Three RCC gene expression profiles (GSE100186, GSE108735, and GSE137836) were downloaded from the Gene Expression Omnibus (GEO) database **(Excel S1)**. GES100186 consisted of eight samples, including four RCC tumor tissues and the corresponding four normal tissues. GSE108735 consisted of 14 samples, including seven RCC tissues and seven corresponding normal tissues. GSE137836 consisted of six samples, including three primary RCC tumor tissues and three metastatic tissues. Differential expression analysis was performed for the normalized data using the limma program; the differentially expressed genes were selected through the following cut-off criteria: lgFC >= 2 and P-value < 0.05. The correlation heat map and volcano map were plotted for the relevant differentially expressed genes; a Venn diagram was drawn using online software (http://bioinformatics.psb.ugent.be/webtools/Venn/) to obtain co-overexpressed circRNAs. The expression of ciRS-7 (hsa_circ_0001946) was visualized using ggpurb.

**Bioinformatics analyses**

To predict the miRNAs that could potentially bind to ciRS-7, first, we obtained the sequence of ciRS-7 from circBank (**Table S1**). Next, we used four online analyses tools: circBank, miRanda, circAtlas, and RNAhybrid to predict the potential miRNAs that could bind to ciRS-7. The prediction results from each database are provided in **Excel S2.**

**RNA sequencing and lab-free** **quantitative analysis**

To identify the target genes of ciRS-7 and the related downstream pathways for RCC, we performed RNA sequencing and label-free quantitative analyses using 786-O cell line transfected with sh-ciRS-7 and control lentiviruses. mRNA expression analysis was performed using Agilent's whole human genome microarray 4×44 K v2 (026652) with monochrome hybridization. This platform includes probes for 34184 human mRNA transcripts. RNA sequencing was performed according to the previously described procedure (8). Label-free quantitative analyses included protein extraction, protein quantification, SDS electrophoresis, reductive alkylation, trypsin digestion, peptide quantification, and liquid-phase tandem mass spectrometry. Sample preparation and microarray hybridization were performed according to the standard protocols in Arraystar (Majorbio, Shanghai, China).

**RNA extraction and quantitative real-time polymerase chain reaction (qRT-PCR)**

Total RNA was extracted from cells or frozen human tissues using TRIzol reagent (TaKaRa, China) according to the manufacturer's instructions. The NE-PER Nuclear and Cytoplasmic Extraction Reagents (Thermo Fisher Scientific, Waltham, USA) was used to isolate the RNA from the nuclear and cytoplasmic fractions. After measuring the concentration and purity of RNA samples using a Nanodrop 2000 spectrophotometer (Thermo Fisher Scientific, USA), reverse transcription was performed using a cDNA kit (R312, Vazyme Biotech, Nanjing, China) to synthesize the cDNA. qRT-PCR was performed using SYBR Green PCR kits (Q141, Vazyme Biotech, Nanjing, China) on the ABI Prism 7500 sequence detection system (Applied Biosystems, USA) and the CT values were determined. All primer sequences are listed in **Table S2**. The relative expression of circRNAs, miRNAs, and mRNA were calculated using the 2^-ΔΔCt^ method; GAPDH and U6 were used as internal standards.

**RNase R and Actinomycin D treatment assay**

The assays were performed according to the previously described protocol([1](#_ENREF_1)). Briefly, RNA was extracted from 786-O and ACHN cells after incubation with RNase R (4 U/mg, Epicenter, USA) for 30 min. 786-O and ACHN cells were treated with 2 µg/ml actinomycin D (HY-17559, Medchemexpress, USA) for 4h, 8h, 12h, and 24h. Then, the relative expression of ciRS-7 and CDR1 was determined by qRT-PCR.

**Fluorescence in situ hybridization (FISH)**

In situ hybridization was performed using specific probes for ciRS-7 and miR-139-3p and the localization of ciRS-7 and miR-139-3p was observed in RCC cells. Briefly, 786-O and ACHN cells were grown on crawl sheets according to the manufacturer's instructions (C10910, RiboBio, Guangzhou, China). After fixation and permeabilization, the cells were probed with cy3-labeled miR-139-3p and fluorescein isothiocyanate (FITC)-labeled ciRS-7 probes overnight at 37°C. The nuclei were then stained with 4,6-diamidino-2-phenylindole (DAPI).

**Cell Counting Kit-8 (CCK-8) and colony formation assays**

Transfected 786-O and ACHN cells were seeded onto 96-well plates (Corning, USA) at a density of 2000 cells per well. After seeding, the cells were allowed to grow for 12h, 24h, 48h, 72h, and 96h, and then, 10µl of CCK8 solution (Yeasen, Shanghai, China) and 100µl of serum-free medium were added to each well and incubated at 37°C in dark for 2h. The optical density (OD) values were determined at 450 nm using a microplate spectrophotometer obtained from BioTek Instruments, Inc. (Winooski, VT, USA).

For colony formation assay, transfected cells were seeded at a density of 500 cells per well onto 6-well plates (Corning, USA) and cultured in complete media for approximately 14 days; cells were replenished with fresh media every five days. After two weeks, the colonies were fixed using formaldehyde and then stained with 0.1% crystal violet (Vicmed, China). These colonies were subsequently photographed and counted.

**Wound healing assay**

The transfected cells were seeded in triplicates onto 6-well plates (Corning, USA). After the cells were attached to the surface and reached 80% confluence, the monolayers were scratched using a 200μL pipette; cell debris was removed by washing thrice with 1×PBS. Subsequently, cells were provided medium supplemented with 2% FBS. Images of cell migration were captured at the same locations at 0 h and 24h after injury, and the wound area was estimated using the ImageJ software (NIH, USA).

**Trans-well invasion and migration assays**

The invasion and migration ability of the cells were assayed using trans-well chambers (8μm pore size, Corning, USA) pre-covered or uncovered with Matrigel (BD Biosciences, USA), respectively. Specifically, transfected cells (5×104) were inoculated in the upper chamber and a culture medium supplemented with 10% FBS was provided in the lower chamber. After 12-24h of incubation, the invading and migrating cells were fixed, stained with 0.1% crystal violet (Vicmed, China), and photographed and counted using an inverted microscope.

**EdU incorporation assay**

The EdU proliferation assay was performed using the Yefluor 488 EdU Imaging Kits (40275, Yeasen, Shanghai, China). Briefly, the transfected cells were inoculated in 96-well plates at a density of 1×10^4^ cells per well. After incubation with 10μM EdU for 2 h, cells were fixed with 4% paraformaldehyde and pro-permeabilized with 0.5% Triton X-100. Subsequently, 0.1ml of Click-iT reaction mixture was added to each well and incubated for 30 min at room temperature in dark before staining for nucleic acids with Hoechst 33342. Images were captured using an Olympus microscope (Olympus, Tokyo, Japan).

**Western blot analysis**

Proteins were extracted by lysing cells on ice with RIPA buffer (Beyotime, China) containing protease inhibitors. The protein concentrations were determined using a BCA protein assay kit (Thermo Fisher Scientific, USA). Protein lysates (50 µg/lane) were separated using 10% sodium dodecyl sulfate-polyacrylamide gels (SDS-PAGE) electrophoresis and transferred onto polyvinylidene fluoride membranes (Merck, USA). The membranes were subsequently blocked for using 5% skim milk for 1h and incubated with primary antibodies overnight at 4°C. Subsequently, the membranes were incubated with respective secondary antibodies (mouse or rabbit) at room temperature for 1h. After washing thrice with PBST, the signals were detected on a Tanon (Shanghai, China) chemiluminescence image analysis system. All antibodies used against the proteins are listed in **Table S3**.

**Biotinylated RNA probe pull-down assay**

After transfecting cells with 3′ end biotinylated miR-139-3p mimics or miR-139-3p NC for 24h, whole-cell lysates were harvested. The lysates were incubated with high-affinity biotin-labeled probes overnight at 4°C on a rotor. Next, the suspension and 50μl of streptavidin magnetic beads (Thermo Fisher Scientific, Inc.) were incubated for 1 h at room temperature and washed twice with wash buffer. Pulled down RNA from the magnetic beads was extracted with TRIzol, and the abundance of ciRS-7 in the bound fraction was assessed by qRT-PCR analysis; each PCR product was analyzed using 2% agarose gel electrophoresis.

**Dual-luciferase reporter assay**

The potential binding site sequences for ciRS-7 and miR-139-3p or miR-139-3p, and TAGLN were obtained, and they were mutated and cloned into the psiCHECK-2 vector (Promega, USA). Cells were inoculated onto 24-well plates, and the luciferase reporter vector (ciRS-7-WT/Mut or TAGLN-WT/Mut) and miR-139-3p mimic or miR-139-3p NC were co-transfected. After 24h of transfection, relative luciferase activity was measured using a dual-luciferase reporter assay system (Promega, USA).

**Colloidal Coomassie staining**

Proteins after WB were visualized by colloidal Coomassie staining. Briefly, after protein separation by SDS-PAGE, the gel was washed with water. The gel was stained with 20 ml of Coomassie staining solution (P0017F, Beyotime, China) at room temperature on a shaker for 30 min. When the target protein bands were visible, the staining solution was discarded and the residual staining solution was washed away using deionized water. The gel was photographed and recorded.

**Preparation of PBAE/si-ciRS-7 nanocomplexes**

PBAE was purchased from RuiXi Biotechnology (Xi'an, China). We prepared nine complexes with different ratios (1, 5, 10, 20, 40, 60, 80, 100, and 120 of PBAE to si-ciRS-7 by weight). Briefly, 5mg PBAE was dissolved in 200μl water to obtain 25μg/μl of PBAE solution. 5nmol si-ciRS-7 was dissolved in 133μl of water to obtain 0.5μg/μl si-ciRS-7 solution. Subsequently, PBAE solutions of different concentrations were vortexed along with a si-ciRS-7 solution for 30 seconds to obtain PBAE/si-ciRS-7 nanocomplexes.

**Quantification of PBAE/si-ciRS-7 nanocomplexes**

0.04µL, 0.2µL, 0.4µL, 0.8µL, 1.6µL, 2.4µL, 3.2µL, 4.0µL, or 4.8µL PBAE (25µg/µL) was added to 187.96µL, 187.8µL, 187.6µL, 187.2µL, 186.4µL, 185.6µL, 184.8µ L, 184.0µL, or 183.2µL of water, respectively, and mixed with 12µL si-ciRS-7 (0.5µg/µL) at room temperature for 20 min. Different mixtures were put in 0.5mL ultrafiltration centrifuge tubes and centrifuged at 12000 rpm for 10 min. The concentration of si-ciRS-7 in the ultrafiltrate was determined and the si-ciRS-7 encapsulation rate was measured.

**Qualitative observation of PBAE/si-ciRS-7 nanocomplexes**

As described above, nanocomplexes of different ratios were prepared. 0.5g agarose was added to 50 ml 1 × TAE buffer, boiled, slightly cooled and, 2.5µL Goldview staining solution was added to it. The gel was poured into the comb gel maker. After the agarose solidified, 20µL of different concentrations of PBAE/si-ciRS-7 nanocomplexes were mixed with 4µL of 6× DNA loading buffer and added into different sparsely dented pores with bare si-ciRS-7 as control. The electrophoresis was performed at 130 V for 15 min. The gel was imaged and all samples were semi-quantified on the Tanon Gel image system (Shanghai, China).

**Potential particle size determination PBAE/si-ciRS-7 nanocomplexes**

0.04µL, 0.2µL, 0.4µL, 0.8µL, 1.6µL, 2.4µL, 3.2µL, 4.0µL, or 4.8µL PBAE (25µg/µL) was added to 17.96µL, 17.8µL, 17.6µL, 17.2µL, 16.4µL, 15.6µL, 14.8µL, 14.0µL, or 13.2µL of water, respectively. Then, 2µL of si-ciRS-7 (0.5µg/µL) was added to and mixed uniformly for 20 min at room temperature. The nanocomplexes so prepared were diluted to 2ml with distilled water. The particle size and spot of the nanocomplexes were determined using a particle size potentiometer (Nano ZS90, Worcestershire, UK).

**Transmission electron microscopy (TEM)**

As described above, the PBAE/si-ciRS-7 nanocomplexes were freshly prepared, dropped into the carbon-coated copper grid, air-dried at room temperature, analyzed, and photographed using TEM (JEOL, Tokyo, Japan).

**Xenograft tumor models**

60 male M-NSG mice aged 4 weeks were purchased from Model Organisms Center, Inc (Shanghai, China). The mice were randomly divided into 12 groups (n=5 per group). All mice were housed in a pathogen-free environment, and all experimental animal operations were performed according to the protocol approved by the Animal Research Ethics Committee of the Shanghai Tenth People's Hospital. Two mice models were designed as follows: a subcutaneous xenograft tumor model and an in vivo lung metastasis model. In addition, PBAE/si-ciRS-7 nanocomplexes treatment and non-treatment groups were formed.

786-O cells stably expressing sh-ciRS-7, control, and OE-ciRS-7 were washed twice with 1×PBS and resuspended in saline. In the subcutaneous xenograft tumor model, 100μl of 5×10^7^ cells mixed with 100μl Matrigel (BD, USA) were injected subcutaneously into the mice. In the lung metastasis model, 200μl of 1×10^6^ cells were injected into the tail vein of mice. The length and width of the mice tumors were measured weekly and the tumor volume was calculated using the following formula: volume (mm^3^) = 0.5 × width^2^ × length. After four weeks, the mice were sacrificed, the lungs and subcutaneous transplanted tumors were excised, and the weight of each tumor was recorded. Lung tissue and some tumor tissues were fixed in 10% buffered formalin. The number of metastatic nodules in the lungs was measured and recorded for subsequent analysis.

786-O cells stably expressing OE-ciRS-7 were collected and subcutaneous xenograft tumor and lung metastasis models were generated according to the above protocol. After five weeks, saline, si-ciRS-7 (10nmol, in vivo-grade cholesterol-conjugated RIG-I siRNA, RiboBio), or PBAE/si-ciRS-7 nanocomplexes (10nmol) were injected intratumorally (50μl) or intravenously (200μl) into the six groups, twice a week for 3 weeks, respectively. Mice were treated as described above.

To simulate the in vivo tumor environment more clearly, we purchased fifteen Balb/c nude mice aged 4 weeks. 1×106 OE-ciRS-7 786-O cells were injected into the right subrenal capsule of each mouse as described previously([2](#_ENREF_2)). Tumor progress was observed using the IVIS imaging system (Calipers, Hopkinton, USA).

**Haematoxylin and eosin (H&E) and immunohistochemical (IHC) staining**

Mice lung tissues were paraffin-embedded and sections with a thickness of 5μm were obtained. These were H&E stained. Mice tumor tissues were fixed with 4% paraformaldehyde, dehydrated using ethanol solution, and then embedded in paraffin wax. The tumor tissues were cut into 4μm sections and immunohistochemistry was performed according to the previously described protocol([3](#_ENREF_3)). The sections were incubated with the appropriate antibodies. Images were captured using a microscope (Leica Microsystems, Germany).

**Statistical analysis**

SPSS 20.0 (IBM, USA), GraphPad Prism 8.3 (San Diego, USA), and R-Studio software (Boston, USA) were used for all statistical analyses. Kaplan-Meier survival curve was used to measure the overall survival (OS) in different ciRS-7 groups, and the results were then evaluated using the log-rank test. Univariate and multivariate Cox regression was used to analyze the effect of ciRS-7 on OS. Two-tailed Student's t-test or χ2 test was used to assess differences between components. Correlation between ciRS-7 and miR-139-3p was evaluated using Spearman's test. For all experiments, P＜0.05 was considered statistically significant.

**References**

1. Dong W, Bi J, Liu H, Yan D, He Q, Zhou Q, et al. Circular RNA ACVR2A suppresses bladder cancer cells proliferation and metastasis through miR-626/EYA4 axis. Mol Cancer. 2019;18(1):95.

2. Gong D, Zhang J, Chen Y, Xu Y, Ma J, Hu G, et al. The m(6)A-suppressed P2RX6 activation promotes renal cancer cells migration and invasion through ATP-induced Ca(2+) influx modulating ERK1/2 phosphorylation and MMP9 signaling pathway. J Exp Clin Cancer Res. 2019;38(1):233.

3. Mao W, Liu S, Wang K, Wang M, Shi H, Liu Q, et al. Cystatin C in Evaluating Renal Function in Ureteral Calculi Hydronephrosis in Adults. Kidney Blood Press Res. 2020;45(1):109-21.

**Table S1.** Full sequence information of ciRS-7.

***>hsa_circ_0001946***

GGTTTCCGATGGCACCTGTGTCAAGGTCTTCCAACAACTCCGGGTCTTCCAGCGACTTCAAGTCTTCCAATAATCTCAAGGTCTTCCAGATAATCCTGAGCTTCCAGAAAATCCACATCTTCCAGACAATCCATGTCTTCCGGACAATCCATGTCTTCCAAGAAGCTCCAAGTCTTCCAGTAAATCAAGTCTTCCAGCAAATCCAGTCTTCCAGCAATTACTGGTCTTCCACCAAATCCAGATCTTCCAGGAAAATCCACGTCTTCCAGGAAATCCATGTCTTCCAATAATTTCAAGGTCTTCCATCAAATACAGATCTTCCAGCTAATCCATGTCTTCCAGAAAAATCTGTGTCTTCCACCAAATCCAAGTCTTCCAGTAAATCTAGTTCTTCCAGAAAAATCTAGATCTTCCAGTCAATCAGTGTCTTCCAGAAAGAAATCCAGGTCTTCCAGTCAATCAGTGTCTTCCAGAAAGAAATCCAGGTCTTCCAGTCAGTCAGTGTCTTCCAGAAAAATCTACGTCTTCCACCAAATCCAGGTCTTCCAGTCAATCCACATCTTCCGGAAAAAATCCAGGTCTTCCAGCCAATATATGTCTTCCTGAAGATCCACGTCTTCCAGAAAATCCATGTCTTCCAGAAAATCCATGTCTTCCAGTAACCTCCCAGTCTTCCAGAAAATCCACGTCTTCCCAACAATCCAAGTCTTCCGGATAATTTGGGTCTTCCTGAAAATCTACGTCTTCCAAAAAAGCCATGTCTTCCAGAAAATCCACATCTTCCAATGGCCTCCAGGTCTTCCAGACTATCCATGTCTTCCAGAAAATCCTTGTCTTCCCTTAAATCTATAGCTTCCAAAAAATCCGGGTCTTCCAGGAAATCCGTGTCTTCCAGCAAGTCCACGTCTTCCAACAAAGCCATGTCTTCCAGACTATCCATGTCTTCCAGAAAATCCTTGTCTTCCCTCAAATCCATAGCTTCCGAAAAATCCAGGTCTTCCAGGAAATCCGTGTCTTCCAGCAAATCCACGTCTTCCAACAAAGCCATGTCTTCCATCAAATTAATGTCTTCCAGCCTACTTGTGTCTTCCAACAAAGGTACGTCTTCCAACAAAGGTACGTCTTCCAACAAAGGTATGTCTTCCAACAAAGGTACGTCTTCCAGAAAATCCACGTCTTCCAACCAAGCCATGTCTTCCAGAAAATCCACGTCTTCCAGAAAATATATGTCTTCCAACTAAGCTACGTCTTCCAACAAATCCATGTCTTCCTATATCTCCAGGTCTTCCAGCATCTCCAGGGCTTCCAGCATCTGCTCGTCTTCCAACATCTCCACGTCTTCCAGCATCTCTGTGTCTTCCAGCATCTTCATGTCTTCCAACAACTACCCAGTCTTCCATCAACTGGCTCAATATCCATGTCTTCCAACGTCTCCAGTGTGCTGATCTTCTGACATTCAGGTCTTCCAGTGTCTGCAATATCCAG

**Table S2.** PCR primer, siRNA and probe sequence.

| **Primes** | **5’-3’ sequence** |
| --- | --- |
| **Primers for PCR** |  |
| ciRS-7-forward | ACGTCTCCAGTGTGCTGA |
| ciRS-7-reverse | CTTGACACAGGTGCCATC |
| CDR1-forward | AGCGCAAAGTGAACAAGAAGAAACAG |
| CDR1-reverse | GGGGCTATGGCAGAAACTCCTCT |
| miR-139-3p-forward | GGAGACGCGGCCCTGT |
| miR-139-3p-reverse | AGTGCAGGGTCCGAGGTATT |
| miR-139-3p-RT Primer | GTCGTATCCAGTGCAGGGTCCGAGGTATTCGCACTGGATACGACACTCCA |
| TAGLN-forward | AGTGCAGTCCAAAATCGAGAAG |
| TAGLN-reverse | CTTGCTCAGAATCACGCCAT |
| GAPDH-forward | CAGGAGGCATTGCTGATGAT |
| GAPDH-reverse | GAAGGCTGGGGCTCATTT |
| U6-forward | CAAATTCGTGAAGCGTTCCATAT |
| U6-reverse | GCTTCACGAATTTGCGTGTCATCCTTGC |
| **siRNAs** |  |
| si-ciRS-7#1 | CTGCAATATCCAGGGTTTC |
| si-ciRS-7#2 | GTCTGCAATATCCAGGGTT |
| si-ciRS-7#3 | GGTTGTCTGCAATCCAG |
| Control | UUCUCCGAACGUGUCACGUTT |
| **RNA pull-down probes** |  |
| ciRS-7 probe | GUGCCAUCGGAAACCCUGGAUAUUG |
| FITC probe | CTTCGTCATCTCCCGAGGATGGAGCGTTCGGGGCCCGTGAACGCTC |
| cy3 probe | TTGTTTGTTGCGTCTGCCTCTCT |
| **FISH probe** |  |
| ciRS-7 probe | GTGCCATCGGAAACCCTGGATATTG |

**Table S3.** Antibodies list.

| **Name** | **Company** | **Number** |
| --- | --- | --- |
| p-PI3K | Cell Signaling Technology | 17366S |
| PI3K | Cell Signaling Technology | 4257S |
| p-AKT | Abcam | ab81283 |
| AKT | Abcam | ab18785 |
| TAGLN | Abcam | ab14106 |
| GAPDH | Abcam | ab8245 |
| Goat Anti-Rabbit IgG H&L (HRP) | Abcam | ab6721 |
| Goat Anti-Mouse IgG H&L (HRP) | Abcam | ab6789 |
|  |  |  |

**Table S4**. The relationship between the expression of ciRS-7 and various clinicopathological variables.

| **Characteristics** | **Total** | **ciRS-7 expression** | | **P value** |
| --- | --- | --- | --- | --- |
|  |  | **Low** | **High** |  |
| Total | 85 | 42 | 43 |  |
| Age categorized, y |  |  |  | 0.952 |
| ≤ 65 | 65 (76.5) | 32 (76.2) | 33 (76.7) |  |
| > 65 | 20 (23.5) | 10 (23.8) | 10 (23.3) |  |
| Gender |  |  |  | 0.187 |
| Male | 64 (75.3) | 29 (69.0) | 35 (81.4) |  |
| Female | 21 (24.7) | 13 (31.0) | 8 (18.6) |  |
| BMI, kg/m^2^ |  |  |  | 0.064 |
| < 25 | 49 (57.6) | 20 (47.6) | 29 (67.4) |  |
| ≥ 25 | 36 (42.4) | 22 (52.4) | 14 (32.6) |  |
| Tumor size, cm |  |  |  | **0.002** |
| ≤ 7 | 70 (82.4) | 40 (95.2) | 30 (69.8) |  |
| > 7 | 15 (17.6) | 2 (4.8) | 13 (30.2) |  |
| T-stage |  |  |  | 0.155 |
| T1/T2 | 63 (74.1) | 34 (81.0) | 29 (67.4) |  |
| T3/T4 | 22 (25.9) | 8 (19.0) | 14 (32.6) |  |
| N-stage |  |  |  | 0.175 |
| N0 | 80 (94.1) | 41 (97.6) | 39 (90.7) |  |
| N1 | 5 (5.9) | 1 (2.4) | 4 (9.3) |  |
| Fuhrman grade |  |  |  | **0.002** |
| I/II | 55 (64.7) | 34 (81.0) | 21 (48.8) |  |
| III/IV | 30 (35.3) | 8 (19.0) | 22 (51.2) |  |

**Table S5.** Univariate and multivariate Cox regression analysis and the relationship between ciRS-7 expression and overall survival.

| **Characteristic** | **Univariate analysis** | | **Multivariate analysis** | |
| --- | --- | --- | --- | --- |
|  | **Hazard Ratio (95% CI)** | **P value** | **Hazard Ratio (95% CI)** | **P value** |
| Age categorized, y |  |  |  |  |
| ≤ 65 (65) | Reference |  |  |  |
| > 65 (20) | 1.19 (0.36-3.88) | 0.775 |  |  |
| Gender |  |  |  |  |
| Male (64) | Reference |  |  |  |
| Female (21) | 0.71 (0.33-1.52) | 0.379 |  |  |
| BMI, kg/m^2^ |  |  |  |  |
| < 25 (49) | Reference |  |  |  |
| ≥ 25 (36) | 0.95 (0.31-2.90) | 0.923 |  |  |
| Tumor size, cm |  |  |  |  |
| ≤ 7 (70) | Reference |  | Reference |  |
| > 7 (15) | 4.34 (1.45-13.01) | 0.009 | - | 0.302 |
| T-stage |  |  |  |  |
| T1/T2 (63) | Reference |  | Reference |  |
| T3/T4 (22) | 5.47 (1.77-16.94) | 0.003 | 4.27 (1.32-13.81) | 0.015 |
| N-stage |  |  |  |  |
| N0 (80) | Reference |  | Reference |  |
| N1 (5) | 1.55 (1.22-2.57) | 0.035 | - | 0.085 |
| Fuhrman grade |  |  |  |  |
| I/II (55) | Reference |  |  |  |
| III/IV (30) | 2.75 (0.92-8.22) | 0.070 |  |  |
| ciRS-7 expression |  |  |  |  |
| Low (42) | Reference |  | Reference |  |
| High (43) | 6.43 (1.42-29.09) | 0.016 | 4.93 (1.06-22.88) | 0.041 |

**Table S6.** Univariate and multivariate Cox regression analysis and the relationship between miR-139-3p expression and overall survival.

| **Characteristic** | **Univariate analysis** | | **Multivariate analysis** | |
| --- | --- | --- | --- | --- |
|  | **Hazard Ratio (95% CI)** | **P value** | **Hazard Ratio (95% CI)** | **P value** |
| Age categorized, y |  |  |  |  |
| ≤ 65 (340) | Reference |  | Reference |  |
| > 65 (173) | 1.77 (1.30-2.43) | <0.001 | 1.66 (1.20-2.29) | 0.002 |
| Gender |  |  |  |  |
| Male (333) | Reference |  | Reference |  |
| Female (180) | 1.11 (0.81-1.54) | 0.518 | 1.07 (0.76-1.51) | 0.693 |
| Grade |  |  |  |  |
| Grade 1/2 (229) | Reference |  | Reference |  |
| Grade 3/4 (276) | 2.74 (1.90-3.97) | <0.001 | 1.75 (1.18-2.61) | 0.006 |
| Stage |  |  |  |  |
| Stage I/II (307) | Reference |  | Reference |  |
| Stage III/IV (203) | 4.00 (2.85-5.61) | <0.001 | 1.84 (0.86-3.89) | 0.114 |
| T-stage |  |  |  |  |
| T1/T2 (325) | Reference |  | Reference |  |
| T3/T4 (188) | 3.36 (2.44-4.64) | <0.001 | 1.13 (0.59-2.18) | 0.707 |
| N-stage |  |  |  |  |
| N0 (225) | Reference |  | Reference |  |
| N1 (17) | 3.00 (1.59-5.66) | <0.001 | 1.44 (0.75-2.76) | 0.227 |
| M-stage |  |  |  |  |
| M0 (403) | Reference |  | Reference |  |
| M1 (78) | 4.21 (3.05-5.82) | <0.001 | 2.27 (1.53-3.38) | <0.001 |
| miR-139-3p expression |  |  |  |  |
| Low (257) | Reference |  | Reference |  |
| High (256) | 0.47 (0.34-0.66) | <0.001 | 0.76 (0.53-1.09) | 0.133 |

**
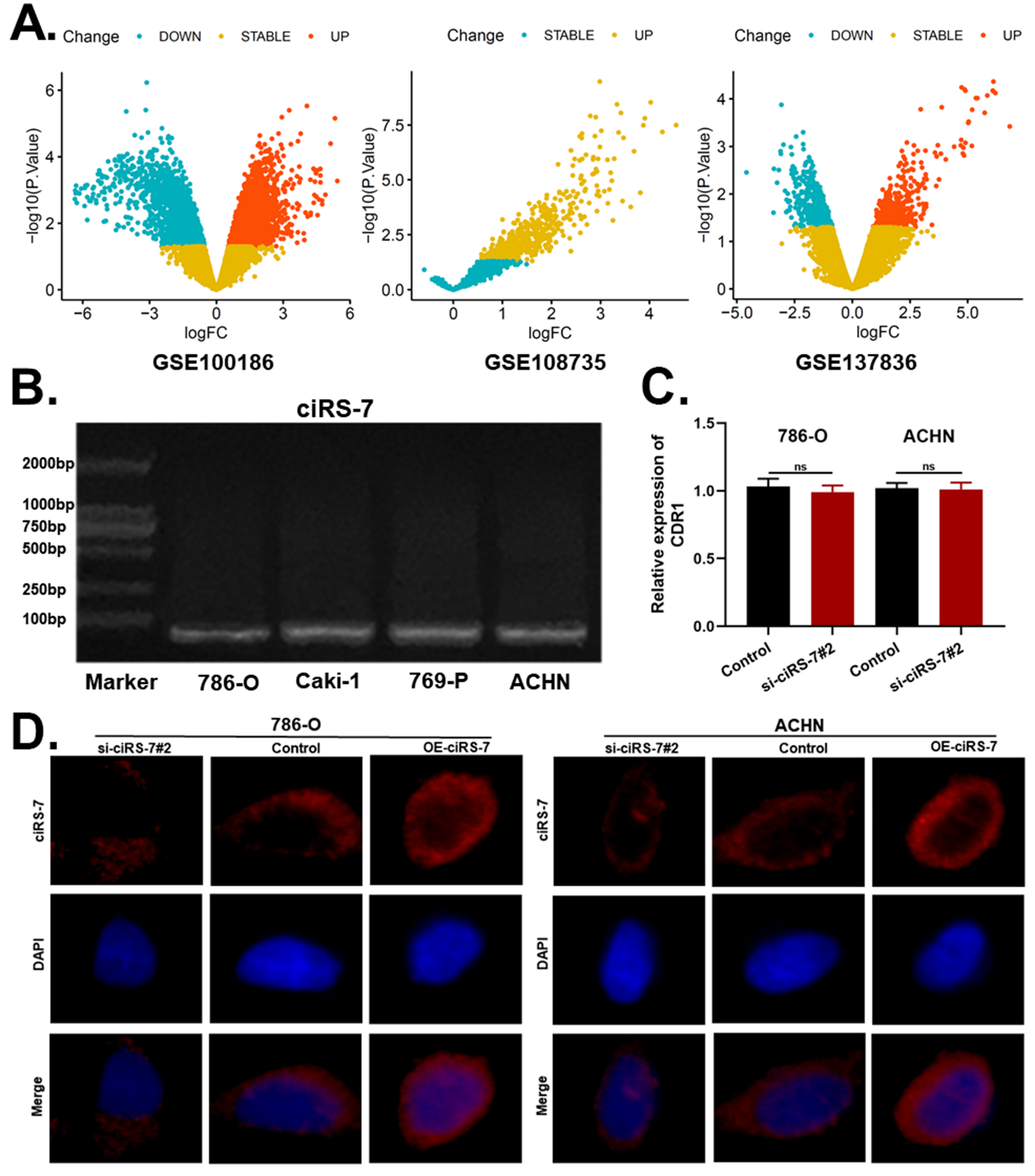
Fig S1**. ciRS-7 was overexpressed in RCC tissues. **A.** Volcano plots analysis of differentially expressed circRNAs in GSE100186, GSE108735 and GSE137836. **B**. Agarose gel electrophoresis of PCR products of ciRS-7 in RCC cell lines. **C**. Relative expression of linear CDR1 was confirmed by qPCR in 786-O and ACHN cell lines transfected with control and si-ciRS-7#2. D**.** Detection of ciRS-7 expression after transfection with the indicated vectors by FISH. Nuclei were stained blue (DAPI), ciRS-7 was stained red.


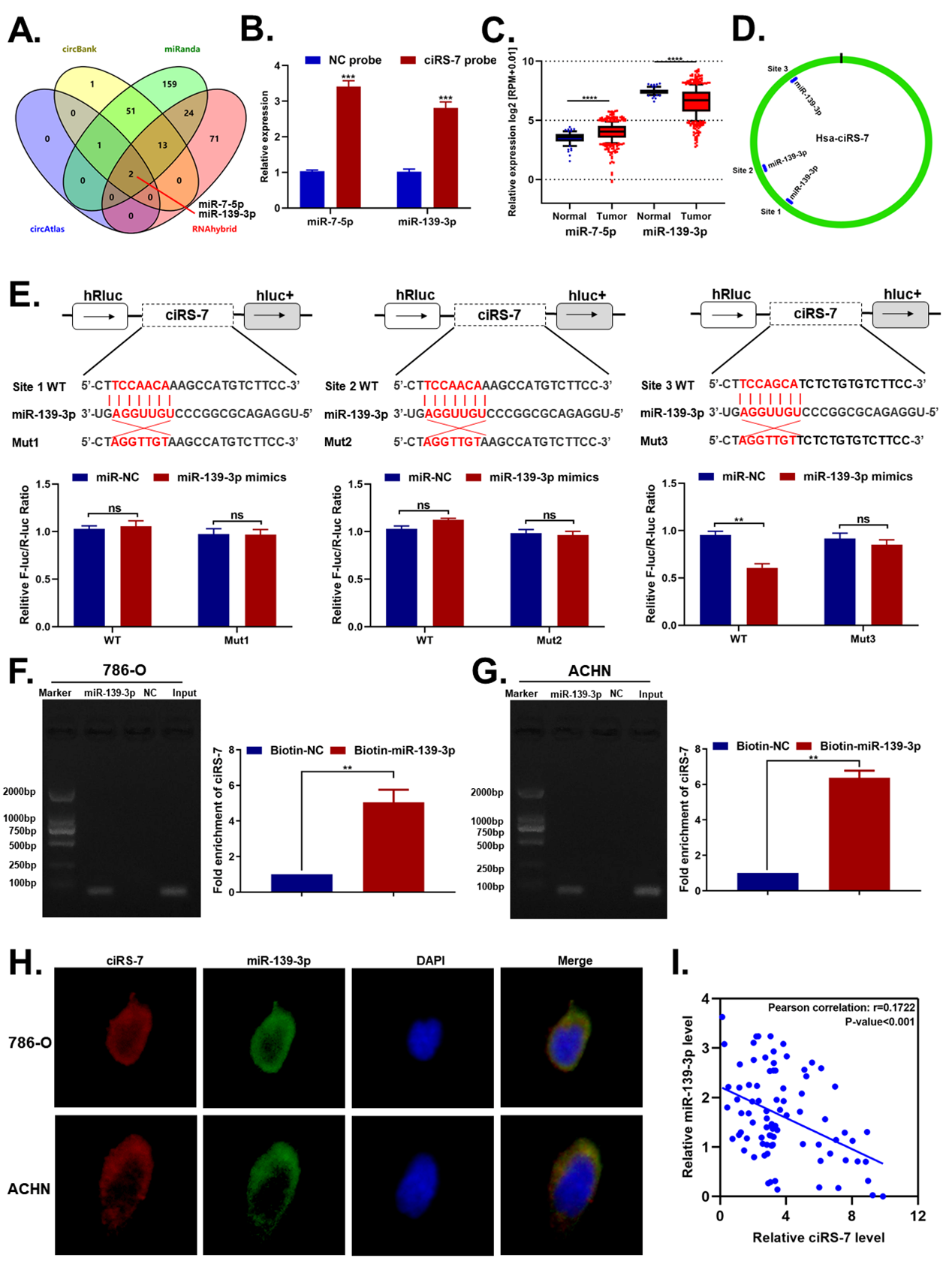


**Fig S2**. ciRS-7 acts as a sponge of miR-139-3p in RCC cells. **A.** Two potential miRNAs absorbed by ciRS-7 were predicted through circBank, miRanda, circAtlas and RNAhybrid. **B**. Relative expression of two miRNAs enriched by ciRS-7 probe lysates was detected by qRT-PCR. **C**. Relative expression of two miRNAs in TCGA RCC database. **D**. Three possible binding sites of miR-139-3p to ciRS-7. **E**. Dual luciferase reporter assay demonstrated that miR-139-3p is a direct target of ciRS-7. **F** and **G**. RNA pull-down assay shown that miR-139-3p is a direct target of ciRS-7. **H**. Detection of colocalization of ciRS-7 and miR-139-3p in cytoplasm by RNA FISH assay. Nuclei were stained blue (DAPI), ciRS-7 was stained red, and miR-139-3p was stained green, **I**. Correlations between ciRS-7 and miR-139-3p expression were found with Pearson’s correlation analysis in RCC tissue samples (n = 85). (***p* < 0.01)


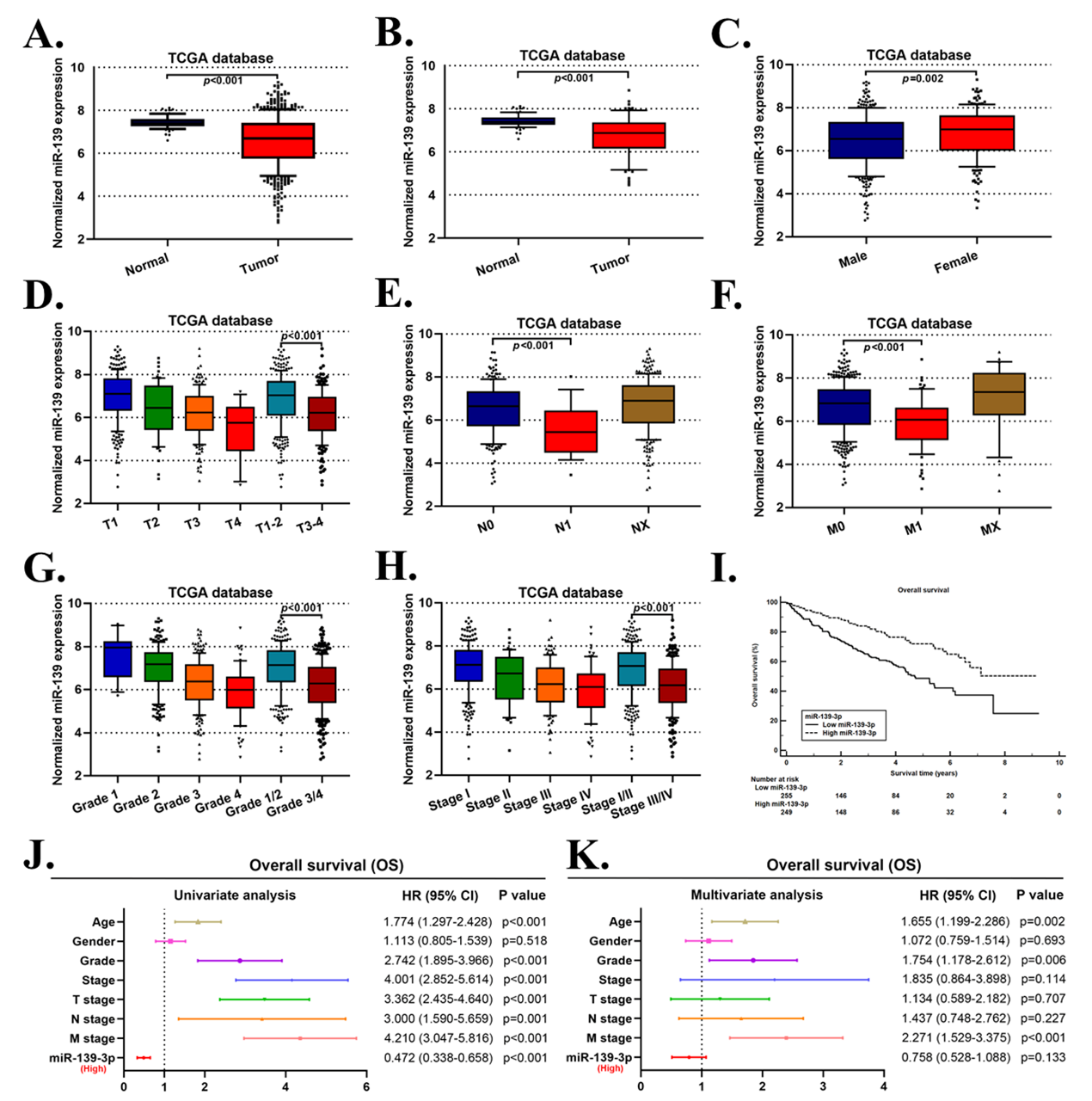


**Fig S3**. miR-139-3p was downregulated in TCGA KIRC database. **A**. Expression of miR-139-3p in normal and tumors tissues of TCGA RCC dataset. **B**. Expression of miR-139-3p in normal and paired tumors tissues of TCGA RCC dataset. **C-H**. Relative expression levels of miR-139-3p in TCGA RCC subgroup: gender (**C**) tumor stage (**D**) lymphatic invasion (**E**) metastasis status (**F**) tumor grade (**G**) and tumor stage (**H**). **I**. Overall survival curve of RCC patients with low and high miR-139-3p expression. **J** and **K**. Univariate and multivariate cox regression analyses of miR-139-3p expression with overall survival in TCGA database.


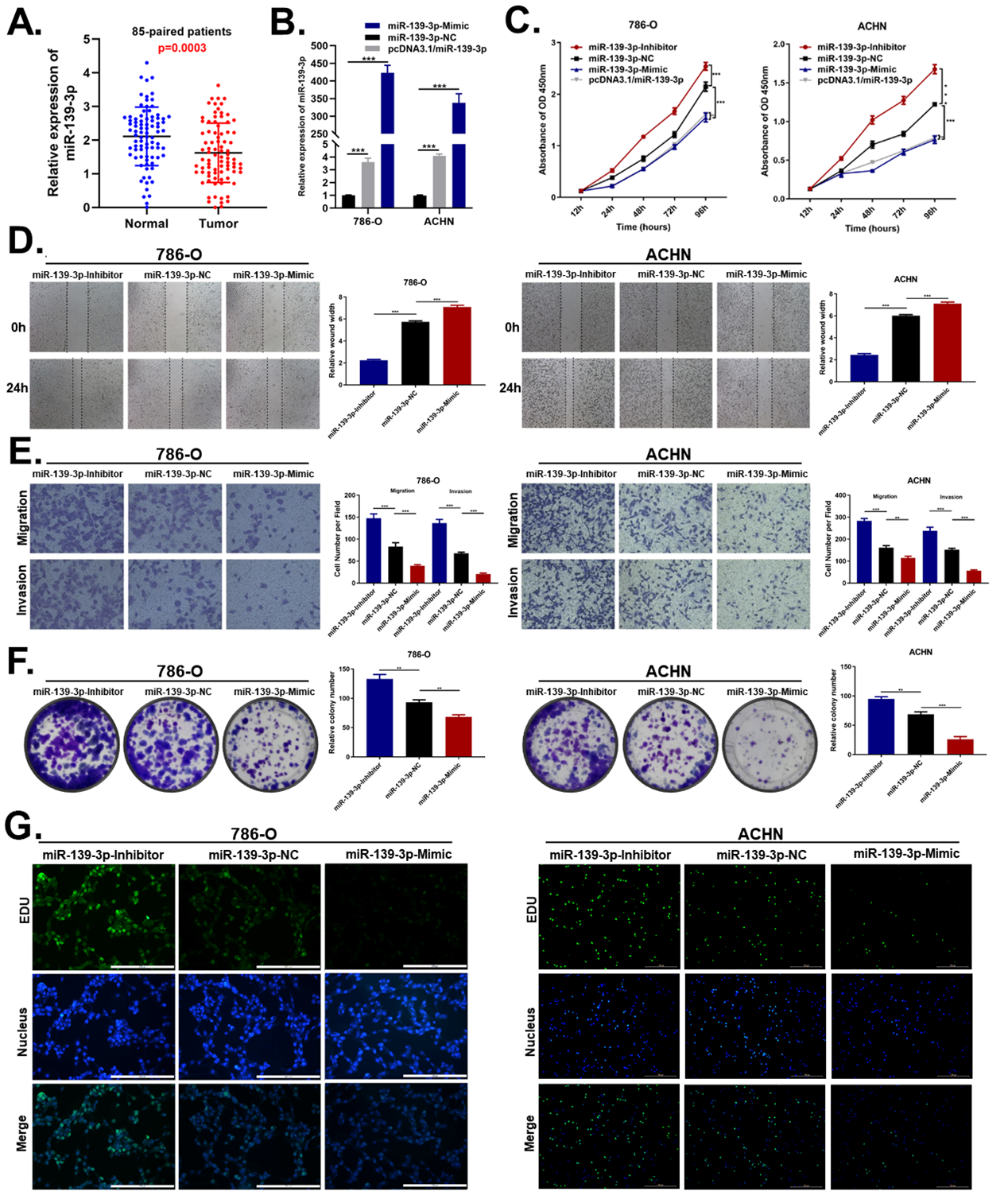


**Fig S4**. miR-139-3p inhibits RCC cell proliferation, migration and invasion *in vitro*. **A**. miR-139-3p had low expression in RCC tumour tissues compared with adjacent normal tissues. **B**. Relative expression of miR-139-3p was confirmed by qPCR in 786-O and ACHN cell lines transfected with miR-139-3p-NC, miR-139-3p-Mimic or pcDNA3.1/miR-139-3p. **C**. Growth curves of 786-O and ACHN cell lines were measured by CCK-8. **D**. Wound healing assay to detect cell migration ability. **E**. Transwell assay to detect cell migration and invasion ability. **F**. Colony formation assay to detect cell migration ability. **G**. Edu assay to detect cell proliferation capacity. (***p* < 0.01, ****p* < 0.001)


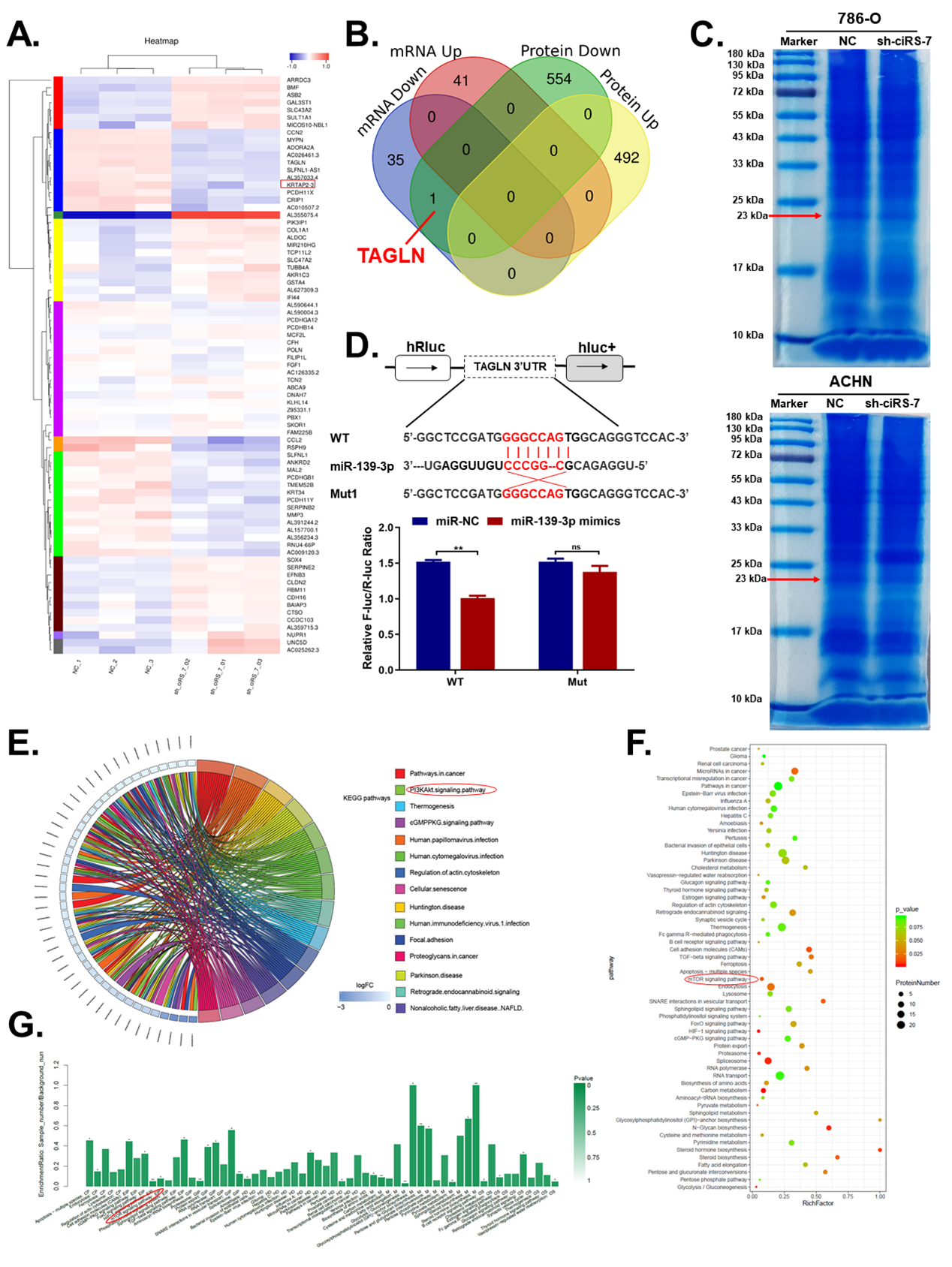


**Fig S5**. TAGLN is a target gene of ciRS-7, and ciRS-7 activates the PI3K/AKT signaling pathway. **A.** Heatmap of RNA-Seq analysis of sh-NC and sh-ciRS-7 cells. Red in the heatmap denotes upregulation, blue denotes downregulation. **B**. Venn diagram showing the number of genes that changes at the transcriptional or protein levels. **C**. colloidal Coomassie detects changes in protein levels in 786-O and ACHN cells. **D**. Dual luciferase reporter assay demonstrated that TAGLN is a direct target of miR-139-3p. **E-G**. Enrichment chord plot (**E**), GO bubble plot (**F**) and KEGG enrichment histogram (**G**) of down-regulated proteins. (***p* < 0.01)

**
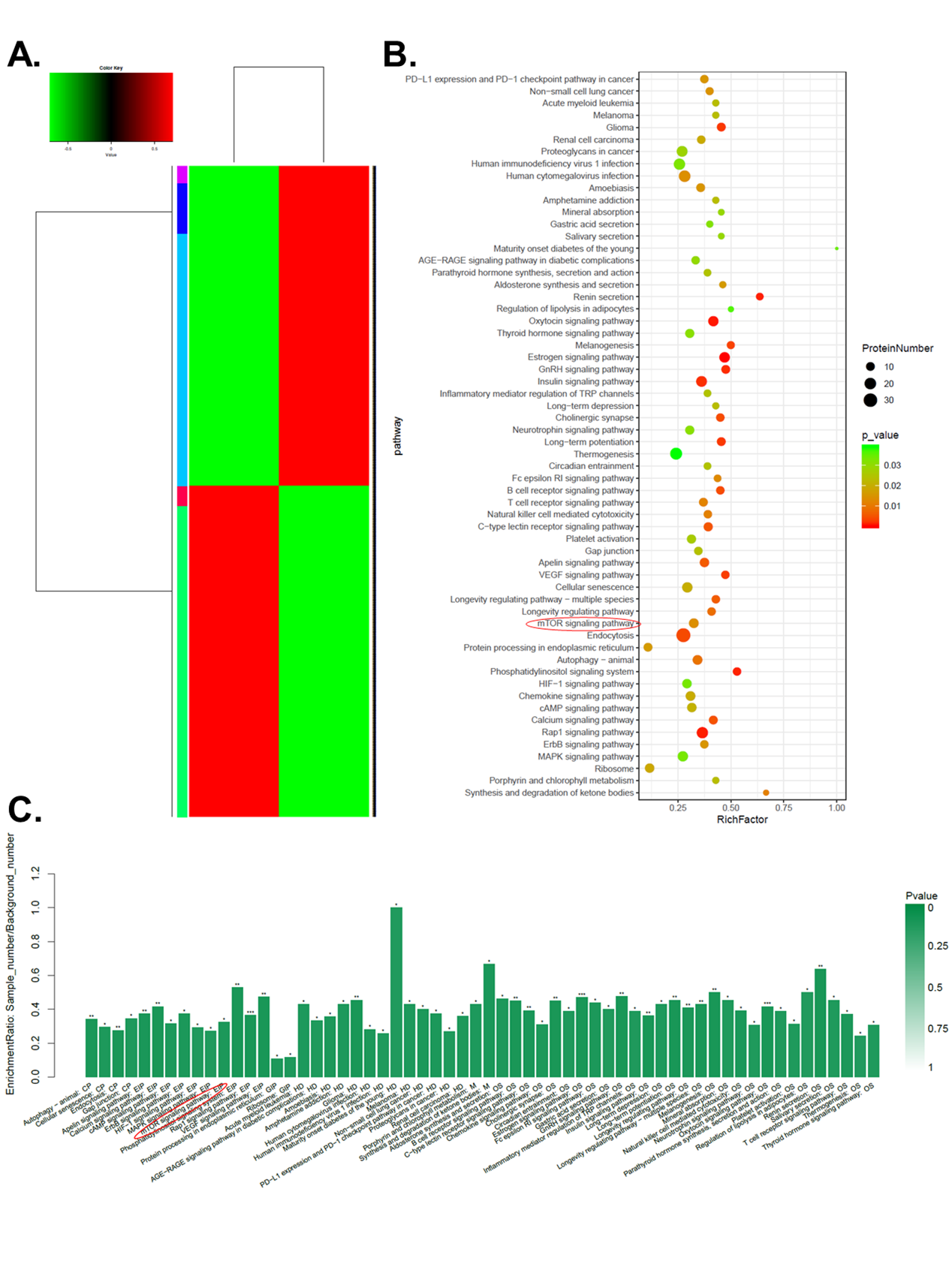
Fig S6.** Sequencing of sh-NC and sh-ciRS-7 cells. **A.** Heatmap of lab-free quantitative of sh-NC and sh-ciRS-7 cells. Red in the heatmap denotes upregulation, green denotes downregulation. **B** and **C**. GO bubble plot (**B**) and KEGG enrichment histogram (**C**) of up-regulated proteins.


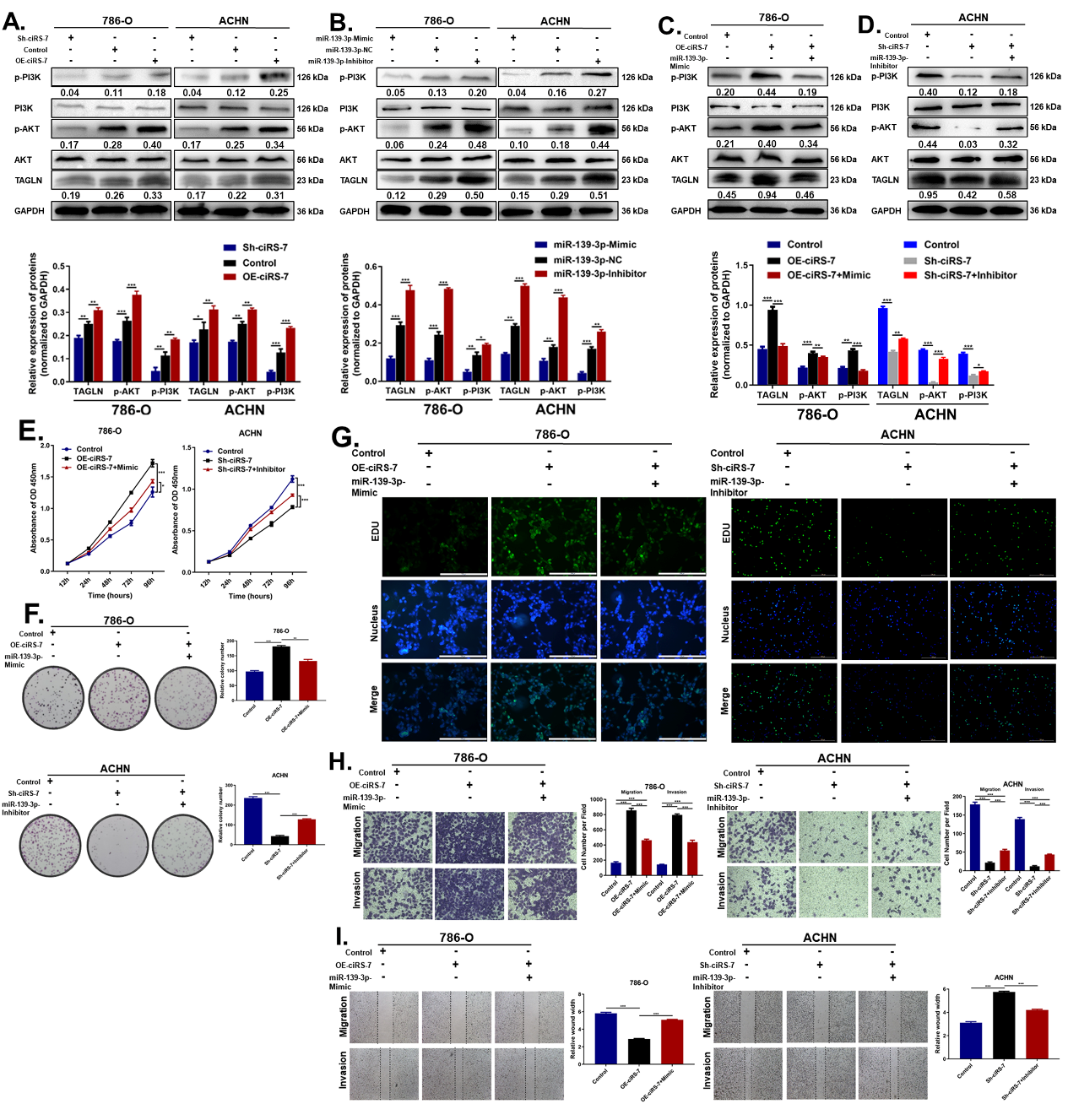


**Fig S7**. ciRS-7 regulating the miR-139-3p/TAGLN axis and activating the PI3K/AKT signaling pathway to promote RCC cell proliferation, migration and invasion. **A.** The expression of TAGLN, p-PI3K and p-AKT were detected by western blot after overexpression or knockdown of ciRS-7. **B**. The expression of TAGLN, p-PI3K and p-AKT were detected by western blot after overexpression or knockdown of miR-139-3p. **C**. Rescue assay of miR-139-3p after overexpression of ciRS-7 in 786-O cells. **D**. Rescue assay of miR-139-3p after knockdown of ciRS-7 in 786-O cells. **E**. Growth curves of 786-O and ACHN cell lines were measured by CCK-8. **F**. Colony formation assay to detect cell migration ability. **G**. Edu assay to detect cell proliferation capacity. **H**. Transwell assay to detect cell migration and invasion ability. **I**. Wound healing assay to detect cell migration ability. (**p* < 0.05, ***p* < 0.01, ****p* < 0.001)


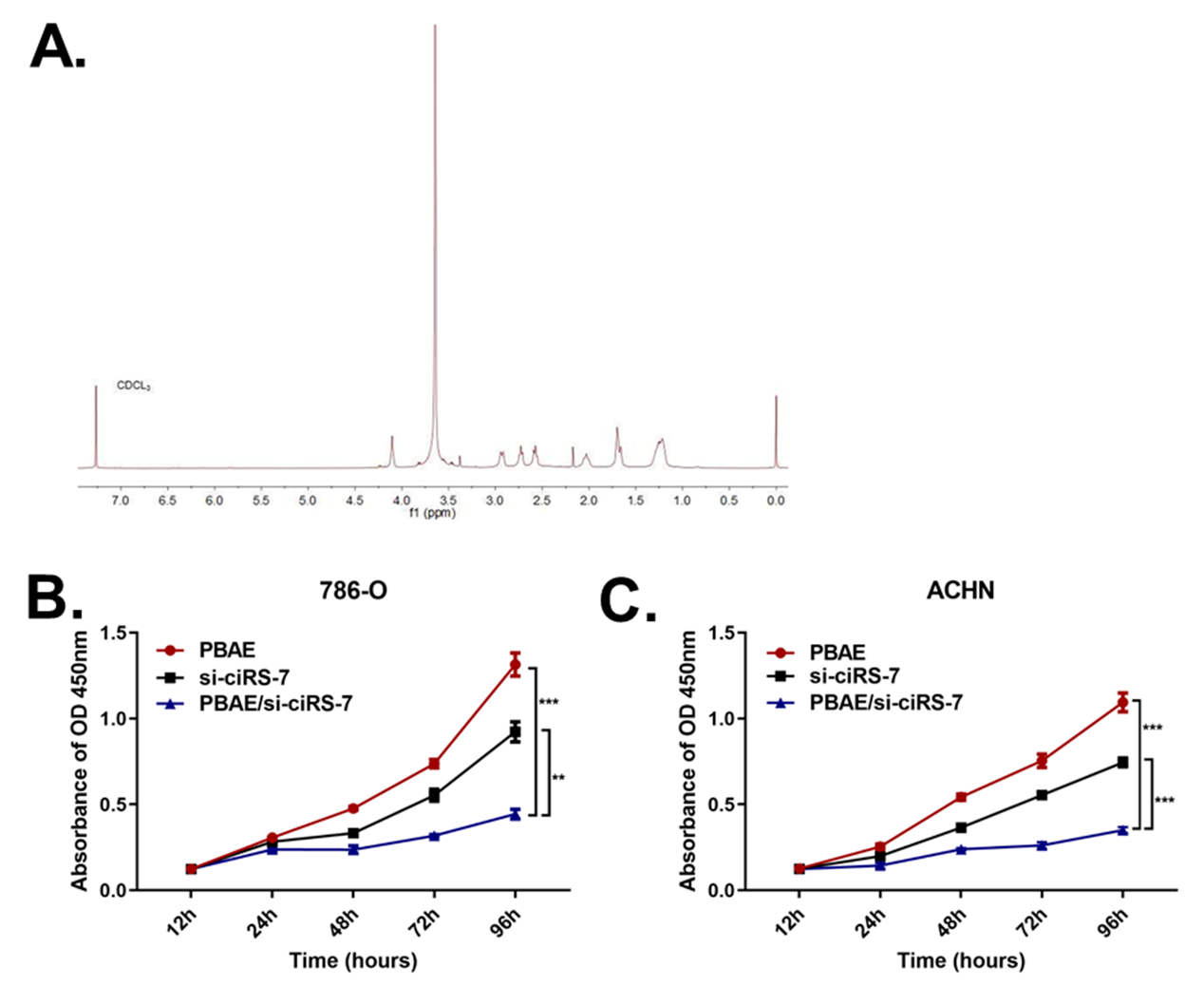


**Fig S8**. Characteristics of PBAE. **A**. The ^1^HNMR analysis of PBAE. **B, C**. Growth curves of 786-O and ACHN cell lines were measured by CCK-8.
